# The cultural adaptation of the go wish card game for use in Flanders, Belgium: a public health tool to identify and discuss end-of-life preferences

**DOI:** 10.1186/s12889-022-14523-9

**Published:** 2022-11-17

**Authors:** Charlèss Dupont, Tinne Smets, Fanny Monnet, Malin Eneslätt, Carol Tishelman, Lieve Van den Block

**Affiliations:** 1 VUB-UGhent End-of-life Care Research Group, Vrije Universiteit Brussel (VUB), 1090 Brussel, Belgium; 2 Department of Learning, Informatics, Management and Ethics, Karolinska Institutet, Stockholm, Sweden; 3 Department of Health, Education and Technology, Luleå University of Technology, Luleå, Sweden; 4 Department of Health Care Sciences, Marie Cederschiöld University College, Stockholm, Sweden; 5 Center for health care economics, informatics and health care research (CHIS). Stockholm Health Care Services (SLSO), Region Stockholm, Stockholm, Sweden

**Keywords:** End-of-life care, Conversations, Advance care planning, Card games, Cultural adaptation, Community engagement

## Abstract

**Background:**

Public health tools like the Go Wish card game from the US, have been found useful to support people in reflecting on their end-of-life preferences, but a cultural adaptation is essential for their success. In the present study, we explore the necessary cultural adaptations to the Go Wish cards by applying an extensive, systematic, and community-engaging negotiating procedure to facilitate the use of the cards in the general population of Flanders, Belgium.

**Methods:**

We used an iterative cultural adaptation process with repeated discussions with various community organizations and representatives of minority and religious groups. After that, the cards were evaluated by 12 healthcare professionals in relation to: linguistic equivalence to the original version, applicability, comprehensibility, and relevance per card. Additional testing with potential users preceded final adjustments.

**Results:**

We found that stakeholders were keen to engage throughout the process of cultural adaptation and we were able to make a range of cultural adaptations for the use of the cards in Flanders. All original statements were rephrased from passive to more active statements. Sixteen out of 36 cards were adjusted to make them more culturally appropriate for use in Flanders, e.g., “to meet with clergy or a chaplain” to “having a spiritual counselor as support.” Three new cards were added: two with statements appropriate to the Belgian patient rights and euthanasia legislation and one extra Wild Card. Potential users (*n* = 33) felt that the cards supported conversations about end-of-life preferences.

**Conclusion:**

By making community engagement a cornerstone of our adaption process, we developed a card set that potential end-users considered a supportive public health tool for reflecting and discussing end-of-life values and preferences. The described process is particularly valuable for culturally adapt interventions, especially given that community engagement in adapting interventions is essential to creating grounded interventions.

**Supplementary Information:**

The online version contains supplementary material available at 10.1186/s12889-022-14523-9.

## Introduction

The European Association for Palliative Care defined advance care planning (ACP) as a process that enables individuals to define goals and preferences for their future medical treatment and care [1]. As noted in this definition, the focus of ACP is shifting from completing advance directives to an ongoing communication and decision-making process [1]. Stimulating individuals to identify end-of-life preferences and wishes have been found to be beneficial for both the patient and their relatives [2–4]. Recent systematic reviews found that individuals who discussed and documented their wishes were more likely to receive their desired end-of-life care [3, 5], and relatives who served as surrogate decision-maker felt it was easier to make decisions since relevant issues had been discussed [4].

Games have been shown to lower reluctance and resistance when discussing potentially uncomfortable topics such as death, dying, and end-of-life care [6] and can thus be helpful in supporting individuals in thinking and talking about ACP and end-of-life values and preferences. One card game that has shown positive results in stimulating discussion in various studies is the American Go Wish card game (developed the mid 90s by Coda Alliance, a U.S.-based non-profit organization) [7–18]. The card game consists of preformulated statements to initiate and support ACP discussions by identifying values and preferences about end-of-life issues and death and dying. The Go Wish cards have been tested in the United States, e.g., with patients on inpatient services [13] and with patients with mild cognitive impairment [11]. Moreover, the Go Wish game has been translated and adapted from its source context to use in other groups, e.g., with parents of children with a life-threatening illness [14], as a teaching tool with medical students [10] and in other cultures, e.g., in Sweden, France, China [7, 8, 15–17, 19–24].

The cultural adaptation of public health tools like the Go Wish card game is likely to be vital for their success [7, 20, 25], as topics like end-of-life care and death and dying are strongly linked to culture and context [26, 27]. Research indicates that culture affects perceptions of health conditions, appropriate treatments, and responses to illness and death [28], aspects of particular importance when thinking and talking about ACP and end-of-life values and preferences. Furthermore, legal contexts can influence people’s thoughts about end-of-life care and death and dying. For instance, in countries where euthanasia was introduced, such as Belgium, there is a substantial relative increase in euthanasia acceptance [29].

Although the literature suggests that cultural adaptation rather than a direct linguistic translation alone is necessary [7, 25], adaptations have not always been performed as comprehensively as might be desired [30]. The target population of the intervention is not always actively involved. However, this has been found to yield improvement in usability and ensure that the adapted version is tailored to the needs of prospective end-users [31–33]. Moreover, it is essential to consider the way researchers, healthcare professionals, and representatives of community organizations are biased in perceptions of how people may think and talk about topics like ACP and end-of-life values and preferences [34, 35]. Therefore, community engagement, where critical actors like individuals, healthcare professionals, and other relevant stakeholders are closely involved throughout the adaptation process, is important for the future success of the adapted intervention [36–38].

In the present study, we explore cultural adaptations to the Go Wish cards determined through an extensive, systematic, negotiated procedure with a wide variety of community stakeholders and pretesting with potential end-users to facilitate the use of the cards in the general population of Flanders, Belgium.

## Methods and materials

### Study context

Belgium is a federal state consisting of three culturally different communities: Flanders, the Dutch-speaking northern part of the country which makes up 56% of the population; Wallonia, the southern French-speaking part of the country with 43.5% of the Belgian population and the German-speaking community in the east where 0.5% of the population lives [39]. In the past years, the Federal authorities have delegated some forms of autonomous responsibility to these communities, e.g. care for older people who are older or disabled, mental health care, primary care, rehabilitation, health promotion, and disease prevention. While we have made three versions of the Go Wish cards, a Dutch, Walloon, and German one, for use in Belgium, we only discuss the Flemish adaptation here because the lessons learned from this adaptation process provided a model for the other translation processes.

### Materials

The original English-language *Go Wish card game* contains 36 cards with 35 single statements that illustrate a behavioral choice or situation based on Steinhauser et al.’s seminal study [40]. The remaining card is a blank ‘wild’ card with no pre-printed statement to allow for other possible issues of importance. The cards can be used in various ways, but original instructions ask users to read through the cards and sort them into three piles: very important, somewhat important, and not important. After that, users are asked to re-examine their “very important” pile, choose their 10 most important cards and rank these from one to 10, with 1 being the most important.

### Study design and procedure

**Fig. 1:**
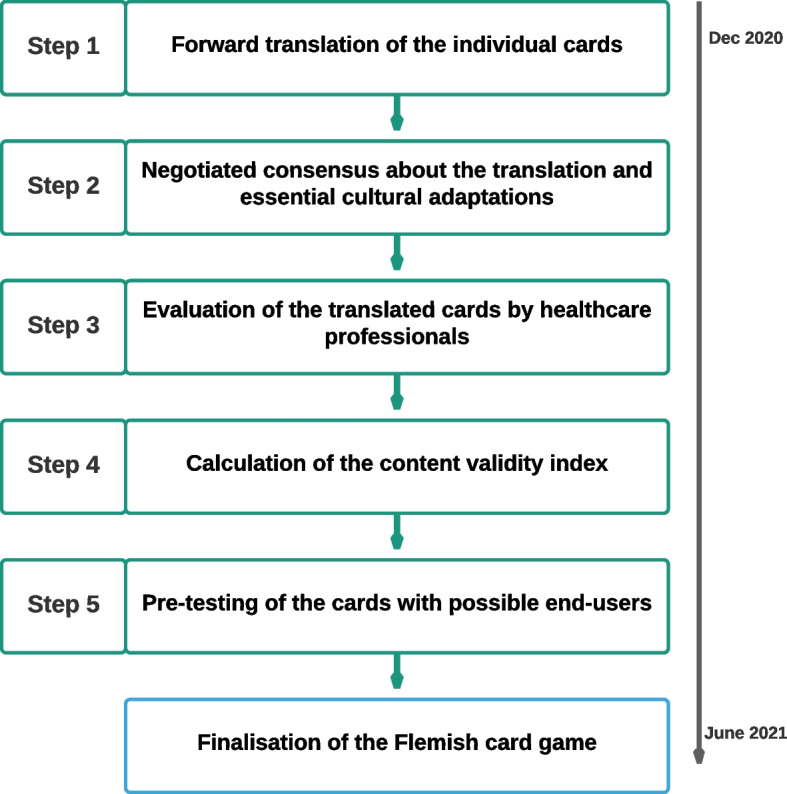
Overview of the cultural adaptation process [41]

We followed the structured, multistep process for cultural adaptation used by McGreevy et al. [41]. Each of the five steps in this process is complementary to the others with the aim of eliciting factors of cultural and linguistic significance through discussion with various stakeholders (Fig. 1).

Additionally, because we aimed to develop a community-based card game for the large and heterogeneous population of all adult individuals with or without life-threatening illness in Flanders, we wanted to ensure that as many perspectives as possible were represented in the cultural adaptation process. To assure inclusion of community perspectives, we formed a reference group consisting of researchers, representatives of the fund Landsbond der Christelijke Mutualiteiten (non-profit health insurance), and representatives of Flemish organizations working with dementia, cancer, family caregivers, end-of-life care, and senior citizens. Representatives were identified via the researchers’ professional networks based on the following criteria: 18 years or above, fluent in Dutch, have a good understanding of English, and have an interest in ACP. This reference group was consulted during steps 2 and after step 5 (Fig. 1) of the cultural adaptation process.

#### Step 1: forward translation

Four researchers each individually completed a preliminary forward translation of all Go Wish cards. Three of these researchers have Dutch as native language, are educated in Flanders, and are fluent in English, while one is a native American English speaker with Dutch as second language. After the translation, one of the four researchers (CD) summarized and compared all four translations and differences were discussed card-by-card by the four researchers in an online meeting until consensus for all cards was reached.

#### Step 2: negotiated consensus about the translation and essential cultural adaptations

In this step, we involved the reference group as well as other strategically selected groups in the process of negotiating consensus on the translation and adaptations. Firstly, the original English-language and Flemish cards translations were sent by email to the members of the reference group. They were asked to comment via email on the translation, the linguistic equivalence of the translated cards to the original cards, the proposed Flemish card game name and to suggest cultural adaptations that they considered essential for use of the cards in Flanders. Cards receiving one or more comments on the translation or on essential cultural adaptations, as well as the proposed Flemish card game name were deliberated upon in online Zoom meetings with the reference group until consensus was reached. After reaching consensus within the reference group as described above, we asked representatives of organisations of people affected by structural vulnerabilities (e.g. ethnic minorities, immigrants, poverty) and representatives of Christianity, Judaism, Islam, the three major religions in Flanders to reflect on the cultural appropriateness of the cards to ensure that the phrases on the cards were open enough, did not exclude or were offensive to minority groups, and avoided systematic bias in relation to particular groups or communities. Representatives were asked to comment on both the translation and cultural appropriateness of each card in one-on-one online meetings with the researcher (CD). This feedback was used to adapt the preliminary version of the Flemish card deck.

#### Step 3: evaluation of the translated cards by healthcare professionals

We evaluated the translated and adapted cards by surveying a variety of healthcare professionals with experience in end-of-life care. Healthcare professionals were recruited by a call via email and social media through the professional networks of the researchers and reference group members, requesting active volunteering with the following inclusion criteria: 18 years or above, fluent in Flemish, good understanding of English, and interest in ACP. When healthcare professionals responded to the recruitment call, the researcher (CD) sent them an email with an information letter and a link to an online consent form and online questionnaire. After consenting, the healthcare professionals could access the questionnaire (example in Additional file 1) and were asked to evaluate the card deck, its linguistic equivalence to the original version, as well as applicability, comprehensibility, and relevance per card on a four-point score (for example for relevance: 1 = Not relevant; 2 = Somewhat relevant; 3 = Quite relevant; 4 = Highly relevant) and through open responses to comment on their scores.

#### Step 4: calculation of the content validity index

We calculated the content validity index (CVI) to measure inter-rater agreement per card for four criteria: linguistic equivalence to the original version, applicability, comprehensibility, and relevance based on the scores derived in Step 3. We calculated the item CVI (I-CVI) for each criterion per card based on the evaluation of the healthcare professionals in Step 3. The I-CVI is the number of healthcare professionals rating a criteria as 3 or 4 on the four-point scale (thus for relevance: 3 = quite relevant; 4 = very relevant) and therefore the proportion of experts giving a positive rating [41–43]. The number of recommended experts to complete the questionnaire is approximately 8 to 12, because when the number of experts grows larger, the probability of chance agreement diminishes [41–43] The minimum recommended I-CVI is 0.78, with 0.90 or higher being considered as an excellent score [41–43]. We calculated the I-CVI scores in Excel by dividing the number of healthcare professionals who rated a card positively, i.e. as quite or highly equivalent, relevant, applicable, or comprehensible by the total number of healthcare professionals who evaluated the card.

#### Step 5: pre-testing of the card game with potential end-users

We assessed and pre-tested the preliminary negotiated Flemish version of the card game with potential end-users. We recruited participants through the networks of the reference group who disseminated a recruitment call via email, their member magazines, newsletters, websites, and social media, using the following inclusion criteria: 18 years or older, fluent in Dutch, able to give informed consent to participate in the study and adequate computer-literacy to participate in an online group discussion. Those interested in participating were asked to send an email to the researcher (CD), who sent further information about the study after verifying participant’s eligibility, and asked them to confirm participation by signing an online informed consent form.

Because of the COVID-19 restrictions, we organized both online and in-person group discussions and participants were given the choice of how they wanted to participate. Participants received an invitation to one of the group discussions which were arranged based on homogeneity within groups (e.g. participant being a family caregiver, patient, being retired, etc.). Participants were invited to the (online) group discussion after providing informed consent. Each participant received the preliminary version of the card game by postal mail and was asked to try the card game before joining the group discussion. During the group discussions, the moderator asked participants to comment on the language used in the cards, give feedback and suggest adaptations when relevant. Participants were also asked to share which cards they considered most important for themselves. At the end of each group discussion, the moderator presented the cards that had an I-CVI score of < 0,78 (based on the feedback of healthcare professionals in step 4) and asked the participants if and how these cards should be adapted. All group discussions were recorded and took place between April and May 2021. Recruitment of participants continued until data saturation was reached, defined as the point at which no remarks emerged.

For each group discussion, participant feedback was summarized both question-by-question and card-by-card, and in relation to the cards each participant considered most important for themselves. Additionally, the observer made detailed notes of participants’ answers, comments, and body language. These detailed notes were analysed inductively to derive suggestions to improve the cards.

#### Finalisation of the Flemish card game

We used the feedback from steps 3 and 5 to make a list of suggested changes to the cards. All cards with a I-CVI < 0,78 (Step 4) were added to the list with the comment that the cards scored relatively low on inter-rater agreement along with the participants’ comments for these cards. This list was sent via email to the reference group with a request to approve or dismiss suggested adaptations per card, and/or suggest other changes. When consensus was reached on all adaptions, the translation and adaptation of the card game was finalized.

### Ethics

Research ethics approval was granted via the Ethical Review Board of Brussels University Hospital of the Vrije Universiteit Brussel (BUN: 1432020000317). All methods were carried out in accordance with relevant guidelines and regulations in the declarationInformed consent was obtained without coercion and only participants who signed an informed consent were included in the study. The cultural adaptation was carried out through a collaboration between the End-of-life Care Research Group of the Vrije Universiteit Brussel and the Landsbond der Christelijke Mutualiteiten (non-profit health insurance).

## Results

As Step 1 entailed the original translation only, we present the results of each step, beginning with Step 2.

### Step 2: negotiated consensus about the translation and essential cultural adaptations

After the four researchers reached consensus, the reference group members (*n* = 11) evaluated the preliminary translation. Fourteen of the 36 cards were accepted as they were, without comments (Table 1). The other 22 cards were discussed one-by-one in the first reference group meeting to seek consensus on the translation and wording, leading to acceptance of the suggested translation of an additional eight cards. For four cards, the reference group proposed slight adaptations to better suit the Dutch way of speaking in Flanders. Ten cards were adjusted for cultural reasons (See Table 1). For example, “to be able to share my accomplishments” was changed to “being able to share my memories” since members of the reference group believed people tend to share memories instead of what they have achieved (Card 9). Also, members of the reference group thought funeral (“uitvaart” in Dutch) was a difficult term, and they therefore changed the card “to have my funeral arrangements made” to “arrange my burial (in Dutch “begrafenis”) in advance” (Card 34). In Flanders the term “begrafenis”, while literally translated as burial, is often colloquially used even for related ceremonies. Lastly, a general comment was that the translation of “to be” into Dutch was too passive and there was consensus in the group to formulate all statements more actively (Table 1).

**Table 1:** Overview of adaptations by card and step

	Ordinal cards	Adaptation after Step 2^1^ (reference group)	Adaptation after Step 2^1^ (organizations structural vulnerabilities and religions)	Adaptations before final version^1^
**1**	To be free of pain	Not being in pain*		
**2**	Not being short of breath	Not being short of breath*		
**3**	To be kept clean	Being neat and clean		Being neat and tidy
**4**	To be free of anxiety	Not being afraid^C^		
**5**	To have human touch	Having physical contact^C^		
**6**	To have my family prepared for my death	That my family is prepared for my death*		
**7**	To die at home	Dying at home*		
**8**	To say goodbye to important people in my life	Being able to say goodbye to my loved ones		
**9**	To remember personal accomplishments	Sharing my memories with others	Being able to share my memories and accomplishments with others	
**10**	To take care of unfinished business with family and friends	Being able to take care of unfinished business with family and friends*		
**11**	To be treated the way I want	Being treated the way I would want to be treated*	Being treated the way I wish to be treated	
**12**	To maintain my dignity	Keeping my dignity*		
**13**	To keep my sense of humour	Keeping my sense of humor*		
**14**	To have close friends near	Being surrounded by good friends*		
**15**	To have someone who will listen to me	Having someone who listens to me*		
**16**	Not being a burden to my family	Not being a burden to my family*		
**17**	To be able to help others	Being able to mean something for someone else	Being able to do something for someone else	
**18**	To be able to talk about what scares me	Being able to talk about what scares me*		
**19**	To have my family with me	Being surrounded by my family*		
**20**	To feel that my life is complete	Feeling that my life is complete*		
**21**	To have a doctor who knows me as a whole person	That the doctor sees me as a whole person*		
**22**	Not dying alone	Not dying alone*		
**23**	To be mentally aware	Be clear-headed^C^		
**24**	To pray	Being able to pray*		
**25**	To meet with clergy or a chaplain	Having a philosophical counsellor	Having a spiritual counselor as support	
**26**	To be able to talk about what death means	Being able to talk about death*		
**27**	To be at peace with God	Be at peace with god	Be at peace with God	
**28**	To have my financial affairs in order	Getting my financial affairs in order*		
**29**	To know how my body will change	Knowing how my body will change*		Knowing how my body and mind will change
**30**	To prevent arguments by making sure my family knows what I want	Avoid discussions by ensuring my family knows what I want^C^		
**31**	To have an advocate who knows my values and priorities	Having someone to represent my values and priorities		Having someone to speak up for what I think is important
**32**	To trust my doctor	Being able to trust my doctor*		
**33**	To have a nurse I feel comfortable with	Having a healthcare professional I feel comfortable with		
**34**	To have my funeral arrangements made	Arrange my burial in advance	Arrange my funeral in advance	
**35**	Not being connected to machines	Not being dependent on machines*		Not being dependent on machines to keep me alive
**36**		A self-chosen end of life^A^		Being able to choose when and how I die^A^
**37**		Being able to record my choices^A^		
**38**	Wild Card	Joker		
**39**		Joker^A,B^		

^1^ Approximated translation to English for this article only. English translation is a forward translation of the final Flemish cards. Additional file 3 elaborates on this Table to include details about reasoning underlying changes

* was accepted as it was (translation in Step 1), with no comments by the reference group on the translation

^A^ card was added

^B^ extra wild card next to the original one

^C^ wording has been slightly adapted to better suit the Dutch way of speaking in Flanders

In the second reference group meeting, we discussed the possibility of adding or removing cards. It was unanimously decided to add a statement related to the legal framework for end-of-life care in Belgium (i.e. the right to choose one’s own end of life, including euthanasia). Although some members wanted to explicitly mention the word ‘euthanasia’, others preferred a more general formulation. Consensus was reached to add two cards: “A self-chosen end-of-life” (card 36) and “being able to record my choices” (card 37). Furthermore, members suggested adding a second “wild card” to give users the opportunity to formulate their own wishes (Table 1). Cards 24 and 27 “to be in peace with god” and “to pray” led to discussions as to whether their religious-focus was appropriate or not. It was decided to keep these cards in the deck, as religion is important for some people when talking about and considering end-of-life preferences. No cards were removed from the deck because all original cards were considered relevant in Flanders. The reference group also decided the card game would be called “*Levenswensen kaartspel”* (In English: “Life Wishes card game”).

Based on feedback from representatives of organizations of people who might be affected by structural vulnerabilities and various religions, translations of six card were revised (see Table 1). Most comments here were related to the use of specific words and formulations. For example, Card 34 had been changed by the reference group as described above, but one religion’s representative opposed the change from ‘uitvaart’ to ‘begrafenis’, arguing that it was less inclusive, as it could be interpreted as excluding cremation. This comment was supported by representatives of organizations of people affected by structural vulnerabilities who often use the term “uitvaart” as funeral insurance is often important to avoid their next of kin having to pay for the funeral. Also, the reference group proposed to write the word “god” in Card 27 without a capital letter to indicate any kind of “God”. However, one representative of a religion disagreed, saying that “God” should be written with a capital letter since in this context it functions as a name. Moreover, whereas the reference group suggested reformulating Card 9, two representatives of people affected by structural vulnerabilities emphasized the importance of accomplishments for people affected by structural vulnerabilities. They suggested that both words (i.e. memories and accomplishments) be included (see Table 1). The representatives of people affected by structural vulnerabilities and various religious groups concluded that the other cards were inclusive enough and would not be offensive for anyone. This feedback was presented to members of the reference group, who agreed with the suggested adjustments and this new version of the deck was accepted.

### Step 3: evaluation of the translated cards by healthcare professionals

The card deck was reviewed by 12 healthcare professionals (General Practitioner *n* = 2, psychologist n = 2, social worker n = 2, psychotherapist *n* = 1, coordinator of local end-of-lifecare center n = 1, head nurse palliative care unit n = 1, care developer n = 1, occupational therapist n = 1, and employee of dementia organization n = 1). All 12 healthcare professionals completed the questionnaire and made suggestions for improvements. For example, seven healthcare professionals commented the word “proper” (which can be translated to “clean”) in Card 3. In Flanders the word “proper” (English “clean”) is more used for an object rather than for personal hygiene. They therefore suggested to adjust it to “verzorgd” (“tidy” or “well-groomed”) since reference is made to being orderly in appearance. Six healthcare professionals found item on Card 9 too long and would have formulated it differently themselves (e.g. reflect on my achievements in life). Also, the new Card 36, “a self-chosen end of life”, was unclear to five healthcare professionals who also indicated that this wording may give an incorrect impression as a self-chosen end-of-life may not always be possible. The adaptions made in this step will be presented under ‘finalization of the cards’.

### Step 4: content validity index

Twenty-eight of the 38 cards had a CVI score of > 0.78 on all four criteria (e.g. linguistic equivalence to the original version, applicability, comprehensibility, and relevance), of which 20/28 scored > 0.90 on all four criteria (Table 2). Three cards scored < 0,78 on linguistic equivalence, eight cards scored < 0,78 on understandability, five cards scored < 0,78 on relevance and six cards scored < 0,78 on applicability in context. Only Card 9 scored < 0,78 on all four criteria.

**Table 2:** Overview of four I-CVI^a^ scores per card

Card Nr.	Step 2 adapted cards	linguistic equivalence	understandability	Relevance	Applicability in context
**1**	Not being in pain	0,92	0,92	0,92	0,92
**2**	Not being short of breath	1,00	1,00	0,91	0,92
**3**	Being neat and proper	0,75*	0,58*	0,83	0,58*
**4**	Not being afraid	1,00	1,00	0,92	0,91
**5**	Having physical contact	0,92	0,92	0,83	0,75
**6**	That my family is prepared for my death	1,00	0,92	0,91	0,83
**7**	Dying at home	1,00	1,00	1,00	1,00
**8**	Being able to say goodbye to my loved ones	1,00	1,00	1,00	1,00
**9**	Being able to share my memories and accomplishments with others	0,67*	0,58*	0,75*	0,75*
**10**	Being able to take care of unfinished business with family and friends	0,83	0,82	0,83	1,00
**11**	Being treated the way I wish to be treated	0,92	0,92	1,00	1,00
**12**	Keeping my dignity	1,00	0,91	1,00	1,00
**13**	Keeping my sense of humor	1,00	1,00	0,92	0,83
**14**	Being surrounded by good friends	0,92	0,92	1,00	1,00
**15**	Having someone who listens to me	1,00	1,00	1,00	1,00
**16**	Not being a burden to my family	0,92	1,00	0,91	1,00
**17**	Being able to do something for someone else	0,75*	0,83	0,92	0,83
**18**	Being able to talk about what scares me	0,91	0,92	1,00	1,00
**19**	Being surrounded by my family	1,00	0,92	1,00	1,00
**20**	Feeling that my life is complete	0,83	0,92	1,00	1,00
**21**	That the doctor sees me as a whole person	0,83	0,83	0,92	0,92
**22**	Not dying alone	1,00	0,92	1,00	1,00
**23**	Be clear-headed	1,00	1,00	1,00	1,00
**24**	Being able to pray	1,00	1,00	0,75*	0,75*
**25**	Having a spiritual counselor as support	0,92	0,75*	1,00	0,83
**26**	Being able to talk about death	1,00	1,00	1,00	1,00
**27**	Be at peace with God	1,00	0,92	0,67*	0,50*
**28**	Getting my financial affairs in order	1,00	0,92	1,00	1,00
**29**	Knowing how my body will change	1,00	0,75*	0,75*	0,75*
**30**	Avoid discussions by ensuring my family knows what I want	0,92	1,00	0,92	1,00
**31**	Having someone to represent my values and priorities	0,83	0,67*	1,00	0,92
**32**	Being able to trust my doctor	1,00	1,00	1,00	1,00
**33**	Having a healthcare professional I feel comfortable with	1,00	1,00	0,83	0,92
**34**	Arrange my funeral in advance	1,00	1,00	1,00	1,00
**35**	Not being dependent on machines	0,83	0,75*	0,92	0,92
**36**	A self-chosen end of life	n/a	0,67*	0,91	0,83
**37**	Being able to record my choices	n/a	0,67*	1,00	0,83
**38/39**	Joker	0,92	1,00	1,00	1,00

^a^ Item Content Validity Index: the inter-rater agreement per card for the four criteria: linguistic equivalence to the original version, applicability, comprehensibility, and relevance

* scored below the minimum recommended I-CVI score of 0.78

### Step 5: pre-testing

In total, 33 individuals between the ages of 35 and 92 participated in the pre-testing of the cards in this step. These individuals received and tested the adapted card set derived from Step 2. Nineteen people participated in six online group discussions: two group discussions were held with cancer survivors (*n* = 4), two with family caregivers (*n* = 6), two with retirees (*n* = 9). Fourteen people participated in four in-person group discussion: two with people from two community centers for people affected by structural vulnerabilities (*n* = 6) and two with nursing home residents (*n* = 8). Four participants in this last group had mild or moderate dementia.

All participants in pre-testing indicated that the cards were user-friendly and assisted them in thinking and talking about end-of-life values and preferences. Twenty-nine of the 33 participants were able to sort the cards into three piles (i.e. very important, important, not important), and choose their top 10 priorities. Only the participants with dementia found ranking the cards into a top 10 list of their priorities too difficult.

The 12 cards with an I-CVI < 0,78 score were discussed in all groups excepting those with nursing home residents. Participants said Cards 31 (*n* = 9), 35 (*n* = 7) and 36 (n = 7) were unclear and suggested rephrasing and simplifying them, but considered them important to include in the deck because of the legal framework in Belgium. For example, several participants said they would want to be connected to machines (Card 35), but not to be kept alive when without prospect of improvement. They therefore suggested rewording Card 35 to “not being dependent on machines that keep me alive”. Moreover, six people affected by structural vulnerabilities said the cards regarding religion and God were the most important cards for them and they were pleased that these cards were in the deck. Two family caregivers of people with dementia and two nursing home residents suggested adding “and mind” to Card 29 as they considered this an important addition for people with cognitive diseases. Sixteen participants mentioned that when testing the card game, they used the wild card to add statements, for example the statement “*my animals are taken care of*”. All participants found that the card set was complete without suggestions for adding or omitting cards. Twelve participants did however suggest revising the name of the card deck *Levenswensen kaartspel (*Life Wishes Card Game) to *Levenswensen kaarten* (Life Wishes Cards) as the word “game” was considered inappropriate.

### Finalisation of the Flemish card game

Based on the feedback of the healthcare professionals in Step 3 and the feedback of participants in Step 5, we made suggestions for adjustments of the 12 cards with an I-CVI score of < 0,78 on one of the four criteria (Table 3). The suggestions for adjustments were submitted to the reference group (via email), who in consensus decided to accept five adaptations. Reasons for the suggested adaptations are summarized in Table 3. An overview of the final version of the Flemish cards can be found in Additional file 3.

**Table 3:** Overview feedback Step 3 and Step 5 and the finalization of the Flemish card game

Card Nr.	Cards	Summery feedback step 3	Summery feedback step 5	Suggested changed	Accepted/not accepted
**1**	Not being in pain				
**2**	Not being short of breath				
**3**	Being neat and proper*	Can be understood as “keeping an object clean” rather than personal hygiene.	Proper is a word used in a “having a clean house” context. Suggest to change to something to underline it is about person hygiene	Being neat and tidy	Accepted
**4**	Not being afraid				
**5**	Having physical contact*	Unclear what kind of physical touch and if users would understand, but there were no problems about if the card was clear.	No comments. Card was perceived as very clear and important.	No changes were suggested since the card was perceived as straightforward and essential by potential users.	
**6**	That my family is prepared for my death				
**7**	Dying at home				
**8**	Being able to say goodbye to my loved ones				
**9**	Being able to share my memories and accomplishments with others*	Need for rewriting this card according to some health professionals as it should be more about “sharing life stories with others” as sharing “accomplishments” is not that important in Flanders.	No comments. Participants choose this card regularly.	No changes were suggested since the card was perceived as essential. Moreover, “share accomplishments” was perceived as important in step 2.	
**10**	Being able to take care of unfinished business with family and friends				
**11**	Being treated the way I wish to be treated				
**12**	Keeping my dignity				
**13**	Keeping my sense of humor				
**14**	Being surrounded by good friends				
**15**	Having someone who listens to me				
**16**	Not being a burden to my family				
**17**	Being able to do something for someone else*	Some healthcare professionals had comments about the translation since they felt this card was about “helping” and found “do something” too vague.	No comments.	No changes were suggested since the card was perceived as clear.	
**18**	Being able to talk about what scares me				
**19**	Being surrounded by my family				
**20**	Feeling that my life is complete				
**21**	That the doctor sees me as a whole person				
**22**	Not dying alone				
**23**	Be clear-headed				
**24**	Being able to pray*	Health professionals considered this card as not relevant for the context and suggested to widen the formulation of this card to “spirituality”	Potential users found this card very important.	No changes were suggested since the card was perceived as important.	
**25**	Having a spiritual counselor as support*	Some healthcare professionals suggested to use another term for “spiritual counselor” as they thought this term was unclear.	Potential users found this card clear	Since the term “spiritual counselor” is accepted and used by the all involved religions, no changes were suggested.	
**26**	Being able to talk about death				
**27**	Be at peace with God				
**28**	Getting my financial affairs in order				
**29**	Knowing how my body will change*	Unclear if it is about during or after life.	For people living with dementia and their family caregivers it was also important to understand how the mind will change.	Knowing how my body and mind will change	Accepted
**30**	Avoid discussions by ensuring my family knows what I want				
**31**	Having someone to represent my values and priorities*	Priorities could be a difficult word to use. Suggestion to change it to important things, values or preferences.	Potential users found the word priorities difficult and suggested to change this card.	Having someone to speak up for what I think is important	Accepted.
**32**	Being able to trust my doctor				
**33**	Having a healthcare professional I feel comfortable with				
**34**	Arrange my funeral in advance				
**35**	Not being dependent on machines*	Unclear what kind of machines. Suggestion to specific that kind of machines.	Unclear what kind of machines. Some potential users said they wanted a machine for comfort but not to keep them alive.	Not being dependent on machines to keep me alive	Accepted
**36**	A self-chosen end of life*	Though all healthcare professionals perceived this card as important, they found the formulation difficult. Some suggested to use the word “euthanasia” of “palliative sedation” as an example in the sentence.	Potential users found this sentence difficult. They suggested to reformulate it in a simpler way.	Being able to choose when and how I die^A^	Accepted
**37**	Being able to record my choices*	Suggestion for more clarification for example “for the end of life”.	No comments. Card was perceived as very clear and important.	No changes were suggested since the card was perceived as clear.	
**38/39**	Joker				

* scored on one of the criteria below the minimum recommended I-CVI score of 0.78

## Discussion

We found that stakeholders were keen to engage throughout the cultural adaptation process, with no difficulties finding healthcare professionals and potential end-users to evaluate the cards. We were able to make a range of cultural adaptations for using the cards in Flanders through the involvement of various community stakeholders. As a result of this collaborative cultural adaptation process, all original Go Wish card statements were rephrased from passive statements (eg “to be” or “to have”) to more active statements. Sixteen cards were adjusted to make them more culturally appropriate for use in Flanders, two new cards were added with statements appropriate to the Belgian legal context in relation to patient rights and euthanasia, and an additional Wild Card was added. The card deck was named *Levenswensen kaarten* (Life Wishes cards).

We found differences among various stakeholder groups to be particularly noteworthy; some cards were found less applicable and relevant by members of the reference group, for example, “to pray”, “to be in peace with God” and “to share my accomplishments”, though these were found very important by representatives of people affected by structural vulnerabilities and various religious groups. This highlights the importance of a cultural adaptation process that involves different perspectives and considers contextual factors in developing interventions [7, 25]. However, there was consensus among members of the reference group from the onset that the Belgian euthanasia law and the right to record one’s own choices should be addressed in the cards. A previous similar cultural adaptation process of the Go Wish cards in Sweden, where euthanasia is not legal, showed that people who described euthanasia as important chose a wild card to formulate a statement on this [7, 8, 16]. In Belgium, the taboo on euthanasia is relatively low and the topic is often part of ACP conversations with physicians [29, 44]. However, the formulation and the terminology have been shown to influence an individual’s preferences [30]. A similar argument was made by the reference group, healthcare professionals, and potential end-users, which led to a decision to formulate the card more broadly than about euthanasia alone.

Most participants in Step 5’s pre-testing of the cards were able to use them with little or no problems with the instructions; however people with dementia found it difficult to prioritize the cards. This is in line with Tishelman et al’s finding that users with cognitive impairment found it difficult to rank “top 10” preferences from their “most important” card choices [7]. Prioritizing preferences requires a high level of abstract reasoning, which may be difficult at times for people with dementia [45]. However, people with dementia have been found to be able to express preferences and wishes, even at a more advanced stage [46, 47] and the use of cards with preformulated statements can support them in this [48] .

Furthermore, this study shows community engagement as a fundamental component in the development of interventions [49, 50]. Throughout the cultural adaptation process we encountered several opposing arguments about possible adaptations but through repeated discussions with various stakeholders like community organizations and citizens, we managed to develop a card set which potential end-users considered a supportive public health tool for reflection and discussion about ACP and end-of-life values and preferences. Hence, in order to normalise conversations about death, dying, and end-of-life care in the community, it is important to involve a wide range of actors like individuals, healthcare professionals and other relevant stakeholders [36, 37]. Community engagement can lead to more grounded interventions that have the potential to be picked up more quickly by the community than interventions developed top-down [36–38]. Moreover, community engagement may increase the capacity to talk about death, dying and end-of-life care which may set the stage to achieve a wider acknowledgement of palliative and end-of-life care as everyone’s responsibility [39–41].

## Strengths and limitations

This study has several strengths. First, from the start of this adaptation process we strived to have strong community engagement which allowed us to include many perspectives using different methods throughout the adaptation process along with a negotiation process used to reach consensus. Second, we used the in-depth multistep process of cultural adaptation developed by McGreevy et al. (41) that was originally developed to translate a taste and smell survey, but has been successfully used in other domains like cardiology [51] and palliative care [52, 53], as well as to adapt the Go Wish cards in Sweden [7]. This process of cultural adaptation including the community engagement can be replicated by others to successfully translate and adapt the Go Wish cards or other culturally-sensitive interventions to their own context. Based on the lessons learned from this adaptation process, we were able to develop a French (Mes souhaits de vie) and German (Meine lebenswünsche) version of the card game for use in the French and German speaking parts of Belgium, respectively.

This study also has some weaknesses. While collaboration with the “Landsbond der Christelijke Mutualiteiten” allowed us to ensure good community engagement, it should be noted that because residents of Belgium are free to choose a non-profit health insurance provider, we may have not reached all potential interested parties. Moreover, even though we used an open sampling strategy to reach many sub-groups in the community, it is difficult to include all possible perspectives in the large and heterogeneous Flemish population. Finally, it is important to recognize that this study does not evaluate the actual use of the card deck. Further research on how people use the cards and whether and what kind of support people need after using the cards is needed.

## Conclusion

Through a collaborative process with strong community engagement in which representatives of Flemish organizations working with dementia, cancer patients, family caregivers, end-of-life care and seniors, a health insurance fund, healthcare professionals and possible end-users were actively involved, we have adapted the English language Go Wish card game for use in Flanders. Sixteen cards from the original card deck were adapted for cultural reasons. Two cards and an extra wild card were added, resulting in a Flemish version with 39 cards. Possible end-users who tested the cards found them to be user-friendly and felt that the cards supported them in thinking and talking about their end-of-life preferences. To normalize thinking and talking about death, dying, and end-of-life care, community engagement in the adaption of interventions is important and can lead to more grounded interventions. Further research on how people use the cards and whether and what kind of support people need after using the cards is needed.

## Supplementary Information

**Additional file 1.** Overview of reasons for cultural adaptations.

**Additional file 2.** Overview Flemish cards.

**Additional file 3.** Exemple questions in questionnaire.

## Data Availability

The datasets generated and/or analyzed during the current study are not publicly available due to restrictions applied to the availability of this data but are available from the corresponding author on reasonable request.

## Abbreviations

ACP: Advance care planning
CVI: Content validity index
I-CVI: Item content validity index

## References

[1] Rietjens JAC, Sudore RL, Connolly M, van Delden JJ, Drickamer MA, Droger M (2017). Definition and recommendations for advance care planning: an international consensus supported by the European Association for Palliative Care. Lancet Oncol.

[2] Piers R, Albers G, Gilissen J, De Lepeleire J, Steyaert J, Van Mechelen W (2018). Advance care planning in dementia: recommendations for healthcare professionals. BMC Palliat Care.

[3] Glass DP, Wang SE, Minardi PM, Kanter MH (2021). Concordance of end-of-life care with end-of-life wishes in an integrated health care system. JAMA Netw Open.

[4] Su Y, Yuki M, Hirayama K (2020). The experiences and perspectives of family surrogate decision-makers: a systematic review of qualitative studies. Patient Educ Couns.

[5] Wendrich-van Dael A, Bunn F, Lynch J, Pivodic L, Van den Block L, Goodman C (2020). Advance care planning for people living with dementia: an umbrella review of effectiveness and experiences. Int J Nurs Stud.

[6] Fernandes CS, Lourenço M, Vale B. Patient card games in palliative care: integrative review. BMJ Support Palliat Care. 10.1136/bmjspcare-2021-003300. Published Online First: 27 October 2021. 10.1136/bmjspcare-2021-003300 34706866

[7] Tishelman C, Eneslätt M, Menkin E, Lindqvist O (2019). Developing and using a structured, conversation-based intervention for clarifying values and preferences for end-of-life in the advance care planning-naïve Swedish context: action research within the DöBra research program. Death Stud.

[8] Eneslätt M, Helgesson G, Tishelman C, Bowers BJ (2020). Exploring Community-Dwelling Older Adults’ Considerations About Values and Preferences for Future End-of-Life Care: A Study from Sweden.

[9] Menkin ES (2007). Go wish: a tool for end-of-life care conversations. J Palliat Med.

[10] Osman H, El Jurdi K, Sabra R, Arawi T (2018). Respecting patient choices: using the ‘go wish’ cards as a teaching tool. BMJ Support Palliat Care.

[11] Siefman M, Brummel-Smith K, Baker S, Edgerton L (2013). Consistency of choices of end-of-life wishes using the “go wish” cards: a comparison of elders with intact cognition and mild cognitive impairment. J Am Geriatr Soc.

[12] Litzelman DK, Inui TS, Schmitt-Wendholt KM, Perkins A, Griffin WJ, Cottingham AH (2017). Clarifying values and preferences for care near the end of life: the role of a new lay workforce. J Community Health.

[13] Lankarani-Fard A, Knapp H, Lorenz KA, Golden JF, Taylor A, Feld JE (2010). Feasibility of discussing end-of-life care goals with inpatients using a structured, conversational approach: the go wish card game. J Pain Symptom Manag.

[14] Potthoff M, Minton M (2017). Go-wish pediatrics: pilot study of a conversation tool in pediatric palliative care. J Pediatr Health Care.

[15] Kroik L, Eneslätt M, Tishelman C, Stoor K, Edin-Liljegren A (2021). Values and preferences for future end-of-life care among the indigenous Sámi. Scand J Caring Sci.

[16] Eneslätt M, Helgesson G, Tishelman C (2021). Same, same, but different? A longitudinal, mixed-methods study of stability in values and preferences for future end-of-life care among community-dwelling, older adults. BMC Palliat Care.

[17] Eneslätt M, Helgesson G, Tishelman C (2021). Dissemination, use, and impact of a community-based, conversational advance care planning intervention: ripple effects of the Swedish DöBra cards. Palliat Care.

[18] Delgado-Guay MO, Rodriguez-Nunez A, De la Cruz V, Frisbee-Hume S, Williams J, Wu J (2016). Advanced cancer patients’ reported wishes at the end of life: a randomized controlled trial. Support Care Cancer.

[19] International Stories – Coda Alliance. [cited 2022 Apr 26]. Available from: https://codaalliance.org/international-stories/

[20] Perin M, Tanzi S, Botrugno C, Craddock C, Menkin E, Peruselli C (2022). Translation and cultural adaptation of the *go wish Game* : thinking about personal values to promote advance care planning. J Palliat Med.

[21] Lefuel P, Bollondi Pauly C, Dufey Teso A, Martin PY, Escher M, Séchaud L (2022). «Jeux sérieux», une nouvelle approche pour aborder le projet de soins anticipé avec les patients dialysés. Nephrol Ther.

[22] Chew YJM, Ang SLL, Shorey S (2021). Experiences of new nurses dealing with death in a paediatric setting: a descriptive qualitative study. J Adv Nurs.

[23] Johansson T, Tishelman C, Eriksson LE, Cohen J, Goliath I (2022). Use, usability, and impact of a card-based conversation tool to support communication about end-of-life preferences in residential elder care – a qualitative study of staff experiences. BMC Geriatr.

[24] Iglesias K, Busnel C, Dufour F, Pautex S, Séchaud L (2020). Nurse-led patient-centred intervention to increase written advance directives for outpatients in early-stage palliative care: study protocol for a randomised controlled trial with an embedded explanatory qualitative study. BMJ Open.

[25] Lunder U, Červ B, Kodba-Čeh H (2017). Impact of advance care planning on end-of-life management. Curr Opin Support Palliat Care.

[26] Gire J (2014). How Death Imitates Life: Cultural Influences on Conceptions of Death and Dying. Online Readings Psychol Cult.

[27] Savard I, Mizoguchi R (2019). Context or culture: what is the difference?. RPTEL..

[28] Gysels M, Evans N, Meñaca A, Andrew E, Toscani F, Finetti S, et al. Culture and End of Life Care: A Scoping Exercise in Seven European Countries. Lucia A. PLoS One. 2012;7(4):e34188. 10.1371/journal.pone.0034188 PMC3317929 22509278

[29] Cohen J, Marcoux I, Bilsen J, Deboosere P, van der Wal G, Deliens L (2006). Trends in acceptance of euthanasia among the general public in 12 European countries (1981–1999). Eur J Pub Health.

[30] Lu A, Mohan D, Alexander SC, Mescher C, Barnato AE (2015). The language of end-of-life decision making: a simulation study. J Palliat Med.

[31] Fischer B, Peine A, Östlund B. The importance of user involvement: a systematic review of involving older users in technology design. Heyn PC. The Gerontologist 2020;60(7):e513–e523. 10.1093/geront/gnz163 PMC7491439 31773145

[32] Shah SGS, Robinson I (2007). Benefits of and barriers to involving users in medical device technology development and evaluation. Int J Technol Assess Health Care.

[33] de Beurs D, van Bruinessen I, Noordman J, Friele R, van Dulmen S (2017). Active involvement of end users when developing web-based mental health interventions. Front Psychiatry.

[34] Callaghan KA, Fanning JB (2018). Managing Bias in palliative care: professional hazards in goals of care discussions at the end of life. Am J Hosp Palliat Care.

[35] Reohr P, Irrgang M, Watari H, Kelsey C (2022). Considering the whole person: a guide to culturally responsive psychosocial research. Psychol Methods.

[36] De Weger E, Van Vooren N, Luijkx KG, Baan CA, Drewes HW (2018). Achieving successful community engagement: a rapid realist review. BMC Health Serv Res.

[37] Okamoto SK, Kulis S, Marsiglia FF, Holleran Steiker LK, Dustman P (2014). A continuum of approaches toward developing culturally focused prevention interventions: from adaptation to grounding. J Prim Prev.

[38] Palmer-Wackerly AL, Krok JL, Dailey PM, Kight L, Krieger JL (2014). Community engagement as a process and an outcome of developing culturally grounded health communication interventions: an example from the DECIDE project. Am J Community Psychol.

[39] Van der Linden N, Roets A (2017). Insights into the Belgian linguistic conflict from a (social) psychological perspective: introduction to the special issue. Psychol Belg.

[40] Steinhauser KE (2000). Factors considered important at the end of life by patients, family, physicians, and other care providers. JAMA..

[41] McGreevy J, Orrevall Y, Belqaid K, Bernhardson BM (2014). Reflections on the process of translation and cultural adaptation of an instrument to investigate taste and smell changes in adults with cancer. Scand J Caring Sci.

[42] Polit DF, Beck CT (2006). The content validity index: are you sure you know what’s being reported? Critique and recommendations. Res Nurs Health.

[43] Polit DF, Beck CT, Owen SV (2007). Is the CVI an acceptable indicator of content validity? Appraisal and recommendations. Res Nurs Health.

[44] De Vleminck A, Batteauw D, Demeyere T, Pype P (2018). Do non-terminally ill adults want to discuss the end of life with their family physician? An explorative mixed-method study on patients’ preferences and family physicians’ views in Belgium. Fam Pract.

[45] Wang HK, Hung CM, Lin SH, Tai YC, Lu K, Liliang PC (2014). Increased risk of hip fractures in patients with dementia: a nationwide population-based study. BMC Neurol.

[46] Wehrmann H, Michalowsky B, Lepper S, Mohr W, Raedke A, Hoffmann W (2021). Priorities and preferences of people living with dementia or cognitive impairment – a systematic review. PPA..

[47] Lepper S, Rädke A, Wehrmann H, Michalowsky B, Hoffmann W (2020). Preferences of cognitively impaired patients and patients living with dementia: a systematic review of quantitative patient preference studies. JAD..

[48] Fried-Oken M, Mooney A, Peters B (2015). Supporting communication for patients with neurodegenerative disease. NRE..

[49] Matthiesen M, Froggatt K, Owen E, Ashton JR (2014). End-of-life conversations and care: an asset-based model for community engagement. BMJ Support Palliat Care.

[50] 1 Recommendations | Behaviour change: individual approaches | Guidance | NICE. NICE; [cited 2022 Apr 27]. Available from: https://www.nice.org.uk/guidance/PH49/chapter/1-Recommendations#recommendation-7-use-proven-behaviour-change-techniques-when-designing-interventions

[51] Ljungberg AK, Fossum B, Fürst CJ, Hagelin CL (2015). Translation and cultural adaptation of research instruments – guidelines and challenges: an example in FAMCARE-2 for use in Sweden. Inform Health Soc Care.

[52] Ammouri AA, Abu Raddaha AH, Tailakh A, Kamanyire J, Achora S, Isac C (2018). Risk knowledge and awareness of coronary heart disease, and health promotion behaviors among adults in Oman. Res Theory Nurs Pract.

[53] Blomberg K, Lindqvist O, Harstäde CW, Söderman A, Östlund U (2019). Translating the patient dignity inventory. Int J Palliat Nurs.

