# Supplementary material for: The cultural adaptation of the go wish card game for use in Flanders, Belgium: a public health tool to identify and discuss end-of-life preferences
Source: BMC Public Health. 2022 Nov 17;22:2110. doi: 10.1186/s12889-022-14523-9 (PMC9672613; doi:10.1186/s12889-022-14523-9)
Supplement: Supplementary file 1 — Additional file 1. Overview of reasons for cultural adaptations. [file 12889_2022_14523_MOESM1_ESM.docx]

Additional file 1 – overview of reasons for cultural adaptations

|  | Ordinal cards | Final Flemish version (English translation)^1^ | Reason for adaptation |
| --- | --- | --- | --- |
| **1** | To be free of pain | Not being in pain | formulated without the expression “to be” |
| **2** | Not being short of breath | Not being short of breath | formulated without the expression “to be” |
| **3** | To be kept clean | Being neat and tidy | By the reference group (Step 2a), it was suggested that "kept" should be modified to "being" (in the moment) and to change the care depended aspect in "to be kept clean". Changed clean to tidy because clean can in Flemish also be interpreted an object being clean, or cleaning something. |
| **4** | To be free of anxiety | Not being afraid | wording has been slightly adapted to better suit the Flemish way of speaking |
| **5** | To have human touch | Having physical contact | wording has been slightly adapted to better suit the Flemish way of speaking |
| **6** | To have my family prepared for my death | That my family is prepared for my death | formulated without the expression “to be” |
| **7** | To die at home | Dying at home | formulated without the expression “to be” |
| **8** | To say goodbye to important people in my life | Being able to say goodbye to my loved ones | formulated without the expression “to be” |
| **9** | To remember personal accomplishments | Being able to share my memories and accomplishments with others | The choice (by reference group in Step 2) has been made to drop achievements and use memories regardless of whether they are something one has achieved because its more about share your life story with others. However, the organizations working around structural vulnerabilities indicated that "achievements" are actually very important to people because they are proud of them and like to share them. We therefore choose to use both words |
| **10** | To take care of unfinished business with family and friends | To be able to settle unfinished business with family and friends* | formulated without the expression “to be” |
| **11** | To be treated the way I want | Being treated the way I wish to be treated | The reference group (Step 2) wanted to reformulate this card to be less forcing. Suggestion for organizations working around minorities: "like to have" to "wish to have. iIs easier to understand. |
| **12** | To maintain my dignity | Keeping my dignity | formulated without the expression “to be” |
| **13** | To keep my sense of humour | Keeping my sense of humor | formulated without the expression “to be” |
| **14** | To have close friends near | Being surrounded by good friends | formulated without the expression “to be” |
| **15** | To have someone who will listen to me | Having someone who listens to me | formulated without the expression “to be” |
| **16** | Not being a burden to my family | Not being a burden to my family | formulated without the expression “to be” |
| **17** | To be able to help others | Being able to do something for someone else | Suggestion by organizations working around minorities (Step 2): this card sounds too much like "hands on" but sometimes people cannot be “hands on” though they can be there for someone else |
| **18** | To be able to talk about what scares me | Being able to talk about what scares me | formulated without the expression “to be” |
| **19** | To have my family with me | Being surrounded by my family | formulated without the expression “to be” |
| **20** | To feel that my life is complete | Feeling that my life is complete | formulated without the expression “to be” |
| **21** | To have a doctor who knows me as a whole person | That the doctor sees me as a whole person | formulated without the expression “to be” |
| **22** | Not dying alone | Not dying alone | formulated without the expression “to be” |
| **23** | To be mentally aware | Be clear-headed | wording has been slightly adapted to better suit the Flemish way of speaking |
| **24** | To pray | Being able to pray | formulated without the expression “to be” |
| **25** | To meet with clergy or a chaplain | Having a spiritual counselor as support | Suggestion by the consulted religions (Step 2): spiritual counselor is umbrella term used by multiple religions |
| **26** | To be able to talk about what death means | Being able to talk about death | formulated without the expression “to be” |
| **27** | To be at peace with God | Be at peace with God | The reference group proposed to write the word “god” without a capital letter because then it could mean any kind of "God”. However, a representative of a religion said that all religions will write “God” with a capital letter since it is about the reverence of sanctity |
| **28** | To have my financial affairs in order | Getting my financial affairs in order | formulated without the expression “to be” |
| **29** | To know how my body will change | Knowing how my body and mind will change | The mind was suggested by family caregivers of people with dementia (Step 5) because according to them this is also something to consider because it is not only the body that can change. |
| **30** | To prevent arguments by making sure my family knows what I want | Avoid discussions by ensuring my family knows what I want | wording has been slightly adapted to better suit the Flemish way of speaking |
| **31** | To have an advocate who knows my values and priorities | Having someone to speak up for what I think is important | Removing specification who because the words "lawyer or representative" have a legal connotation in Belgium and the reference group (Step 2) wanted to avoid this connotation. Also, changed priorities to “what I think is important” because according to organizations working with people in structural vulnerabilities priorities is a difficult word |
| **32** | To trust my doctor | Being able to trust my doctor | formulated without the expression “to be” |
| **33** | To have a nurse I feel comfortable with | Having a healthcare professional I feel comfortable with | To be more inclusive nurse was adjusted to caregivers because patients interact with many types of caregivers |
| **34** | To have my funeral arrangements made | Arrange my funeral in advance | The reference group though funeral (“uitvaart” In Dutch) was a difficult term and changed the card to “arrange my burial (in Dutch “begrafenis”) in advance”. In Flanders we often use the term “begrafenis” for a funeral, though when people use this term (which can be translated to “burial”), they also mean the related ceremonies (thus not only the interment or entombment). But a representative of a religion said that a “burial” is not an inclusive term and according to and representatives of people affected by structural vulnerabilities said the word “funeral” (in Dutch “uitvaart”) is often known by people because they associate it with their insurance. |
| **35** | Not being connected to machines | Not being dependent on machines to keep me alive | Unclear for some healthcare professionals and participants in the pre-testing what exactly those machines are (Step 3, 4 and 5). Added the clarification. |
| **36** | Wild Card | Being able to choose when and how I die | Added because of the legislation in Belgium. Only the initial wording (self-chosen end of life) was not clear to healthcare professionals and participants in the pre-testing so clarified and made wording easier |
| **37** |  | Being able to record my choices |  |
| **38** | Wild Card |  |  |
| **39** |  | Extra Wild Card |  |
