# Supplementary material for: The cultural adaptation of the go wish card game for use in Flanders, Belgium: a public health tool to identify and discuss end-of-life preferences
Source: BMC Public Health. 2022 Nov 17;22:2110. doi: 10.1186/s12889-022-14523-9 (PMC9672613; doi:10.1186/s12889-022-14523-9)
Supplement: Supplementary file 2 — Additional file 2. Overview Flemish cards. [file 12889_2022_14523_MOESM2_ESM.docx]

Additional file 2 – Overview Flemish cards

|  | Go Wish card game | Levenswensen kaarten |
| --- | --- | --- |
| **1** | To be free of pain | Geen pijn hebben |
| **2** | Not being short of breath | Niet kortademig zijn |
| **3** | To be kept clean | Netjes en verzorgd zijn |
| **4** | To be free of anxiety | Geen angst hebben |
| **5** | To have human touch | Lichamelijk contact hebben |
| **6** | To have my family prepared for my death | Dat mijn familie voorbereid is op mijn dood |
| **7** | To die at home | Thuis sterven |
| **8** | To say goodbye to important people in my life | Afscheid kunnen nemen van mijn dierbaren |
| **9** | To remember personal accomplishments | Mijn herinneringen en verwezenlijkingen kunnen delen met anderen |
| **10** | To take care of unfinished business with family and friends | Onafgeronde zaken met familie en vrienden kunnen bespreken |
| **11** | To be treated the way I want | Dat er met mij wordt omgegaan zoals ik dat wens |
| **12** | To maintain my dignity | Mijn waardigheid kunnen behouden |
| **13** | To keep my sense of humour | Mijn gevoel voor humor kunnen bewaren |
| **14** | To have close friends near | Omringd zijn door goede vrienden |
| **15** | To have someone who will listen to me | Iemand hebben die naar mij luistert |
| **16** | Not being a burden to my family | Mijn familie niet tot last zijn |
| **17** | To be able to help others | Nog iets voor iemand anders kunnen betekenen |
| **18** | To be able to talk about what scares me | Kunnen praten over wat mij bang maakt |
| **19** | To have my family with me | Omringd zijn door mijn familie |
| **20** | To feel that my life is complete | Het gevoel hebben dat mijn leven compleet is |
| **21** | To have a doctor who knows me as a whole person | Een arts hebben die mij goed kent als persoon |
| **22** | Not dying alone | Niet alleen sterven |
| **23** | To be mentally aware | Helder van geest zijn |
| **24** | To pray | Kunnen bidden |
| **25** | To meet with clergy or a chaplain | Een spiritueel begeleider als steun hebben |
| **26** | To be able to talk about what death means | Kunnen praten over de dood |
| **27** | To be at peace with God | Vrede hebben met God |
| **28** | To have my financial affairs in order | Mijn financiële zaken op orde hebben |
| **29** | To know how my body will change | Weten hoe mijn lichaam en geest zullen veranderen |
| **30** | To prevent arguments by making sure my family knows what I want | Discussies voorkomen door te zorgen dat mijn familie weet wat ik wil |
| **31** | To have an advocate who knows my values and priorities | Iemand hebben die opkomt voor wat ik belangrijk vind |
| **32** | To trust my doctor | Mijn arts kunnen vertrouwen |
| **33** | To have a nurse I feel comfortable with | Zorgverleners hebben bij wie ik mij op mijn gemak voel |
| **34** | To have my funeral arrangements made | Mijn uitvaart op voorhand regelen |
| **35** | Not being connected to machines | Niet afhankelijk zijn van machines die mij in leven houden |
| **36** |  | Zelf kunnen kiezen wanneer en hoe ik sterf |
| **37** |  | Mijn keuzes kunnen vastleggen |
| **38** | Wild Card | Joker |
| **39** |  | Joker |
