# Supplementary material for: The cultural adaptation of the go wish card game for use in Flanders, Belgium: a public health tool to identify and discuss end-of-life preferences
Source: BMC Public Health. 2022 Nov 17;22:2110. doi: 10.1186/s12889-022-14523-9 (PMC9672613; doi:10.1186/s12889-022-14523-9)
Supplement: Supplementary file 3 — Additional file 3. Exemple questions in questionnaire. [file 12889_2022_14523_MOESM3_ESM.docx]

Additional file 3 – exemple questions in questionnaire

| Card 1 - To be free of pain | | | | |
| --- | --- | --- | --- | --- |
| Does this Dutch translation for Flanders mean the same thing as the original card? | - Not equivalent | - Somewhat equivalent | - Quite equivalent | - Highly equivalent |
| Do you find this card comprehensible? | - Not comprehensible | - Somewhat comprehensible | - Quite comprehensible | - Highly comprehensible |
| Do you find this card is applicable to the Flemish context? | - Not applicable | - Somewhat applicable | - Quite applicable | - Highly applicable |
| Do you find this card relevant to the card set? | - Not relevant | - Somewhat relevant | - Quite relevant | - Highly relevant |
| Do you have any comments or concerns about this card? |  | | | |
